# Parenthood and life satisfaction in older age: examining the moderating role of social norms and economic vulnerability

**DOI:** 10.1007/s10433-025-00853-1

**Published:** 2025-04-04

**Authors:** Matthias Pollmann-Schult

**Affiliations:** Siegen University, Siegen, Germany

**Keywords:** Parenthood, Life satisfaction, Subjective well-being, Cross-country comparison

## Abstract

Research on the relationship between parenthood and life satisfaction has revealed mixed results, with older parents reporting higher life satisfaction than older nonparents in some countries but not in others. This study investigates whether the link between parenthood and life satisfaction among individuals aged 60 years and older systematically varies across countries. Drawing on the theoretical premise that country-specific factors influence both the benefits of parenthood and the psychological costs of childlessness, the study examines the roles of pronatalist norms, filial elder-care norms, and the economic conditions of older individuals in shaping the life satisfaction gap between parents and nonparents. The study analyzes European Social Survey data on 114,513 individuals aged 60 years and older in 32 European countries using multilevel regression models. The results show that the life satisfaction gap between parents and nonparents is positively related to the strength of pronatalist norms and the level of economic vulnerability among older people. In contrast, elder-care norms are not uniformly linked to the magnitude of the life satisfaction gap. However, a particularly large life satisfaction gap was observed in countries with both high levels of economic vulnerability and strong elder-care norms. These findings suggest that the extent to which parenthood affects the life satisfaction of older individuals strongly depends on societal context.

## Introduction

Fertility rates in Europe have significantly declined since the mid-1960s (Schumacher et al. 2024), resulting in a growing proportion of older individuals without children (Sobotka 2017). This demographic shift has raised concern regarding the subjective well-being (SWB) of childless older individuals (Verweij and Keizer 2021), as children often provide emotional and instrumental support to their aging parents (Broese van Groenou and De Boer 2016).

While theoretical frameworks such as the value-of-children approach (Hoffman and Hoffman 1973; Nauck 2007) propose that older individuals with adult children experience greater SWB than their childless counterparts, empirical evidence on the extent and nature of this relationship is highly inconsistent. Dykstra and Wagner (2007), Hank and Wagner (2013), Huijts et al. (2013), and Simon and Caputo (2019) observed higher levels of SWB among parents compared with childless individuals, while Gibney et al. (2017) have suggested that parents of adult children experience lower levels of SWB than childless individuals. However, the majority of empirical studies have found no significant differences in SWB between older parents and their childless counterparts (e.g., Bures et al. 2009; Dykstra and Keizer 2009; Evenson and Simon 2005; Hansen et al. 2009; Pudrovska 2008).

These inconsistent findings have led researchers to pay closer attention to the relevance of societal context for the SWB of parents and nonparents. Multi-country studies have indicated that conflicting results from single-country studies may be due to the diverse social contexts in which the studies were conducted (see, Hansen 2021). Indeed, it appears that parenthood is more positively associated with SWB in southern and Eastern Europe than in northern and Western Europe (e.g., Gibney et al. 2017; Grundy et al. 2017; Margolis and Myrskylä 2011). These cross-country differences are typically attributed to variations in social policies and cultural norms. However, most multi-country studies do not explicitly explore which specific country-level characteristics generate cross-country differences. To the best of my knowledge, only three previous studies (Huijts et al. 2013; Mair 2013; Neuberger and Preisner 2018) have examined the moderating role of country characteristics in the association between parenthood and various indicators of subjective well-being. Huijts et al. (2013) and Mair (2013) explored whether cross-national differences in family culture, social cohesion, and public pension expenditure account for differences in depressive symptoms between parents and nonparents, while Neuberger and Preisner (2018) examined whether differences in public expenditure on older people correlate with cross-national disparities in quality of life. Overall, these studies provide inconclusive evidence, possibly due to the limited number of countries in the data sets used by these studies (14, 19, and 24 countries, respectively).

The present study analyzed differences in the relationship between parenthood and life satisfaction among older individuals across 32 European countries. It explores whether this variation can be attributed to cross-country differences in filial elder-care norms, pronatalist norms, and the economic vulnerability of older people. To this end, I used data from the European Social Survey (ESS), which includes information on 114,513 individuals aged 60 years and older.

## Background

Research on SWB in older age commonly posits that older parents experience higher levels of well-being than their childless counterparts (see, Hansen 2012). This view is theoretically grounded in the value-of-children approach (Hoffman and Hoffman 1973; Nauck 2007) and related concepts. Although the value-of-children approach primarily focuses on minor children, it can be extended to adult children. As Hoffman (1975) outlined, parenthood provides a broad range of benefits that enhance subjective well-being (SWB), spanning from “economic utility” to the fulfillment of emotional needs. Similar to marital relationships (Finkel et al. 2014), expectations surrounding the parent–child relationship have likely evolved and vary across different contexts. In their study on parenthood and SWB in old age, Neuberger and Preisner (2018) considered the different rewards of parenthood by contrasting the supportive benefits assumption, which emphasizes the role of adult children in providing informal support, with the emotional benefits assumption, which highlights their role as a key source of social connection and interaction. This perspective also suggests that emotional and instrumental benefits interact in their influence on SWB, and that emotional benefits play a greater role in contexts where older people are not reliant on instrumental family support (Neuberger and Preisner 2018).

Individual-level studies have identified filial social support as a key mechanism linking parenthood to SWB in old age. Adult children play a central role in the social networks of their aging parents (Hank 2007) and provide both emotional and instrumental support (Kjær and Siren 2021; Wu et al. 2023), which in turn enhances psychological well-being (Berkman et al. 2000; Ferlander 2007; Pinquart and Sörensen 2000). The disadvantages (or costs) of childlessness not only stem from the absence of filial support; they also result from psychological burdens associated with being childless. Before the 1960s, childlessness was largely unintentional (see, Hansen 2021), mostly resulting from factors such as remaining unmarried, marrying later, or experiencing infertility. Remaining childless was often signified a deviation from the anticipated life trajectory (Hagestad and Call 2007; Tanaka and Johnson 2016). As a consequence, older childless people may feel that they have not achieved their personal goals and expectations (see, Buchinger et al. 2022), possibly leading to feelings of loss and failure, which can ultimately reduce SWB (Alexander et al. 1992).

In addition to the individual-level factors outlined above, the benefits of parenthood and the costs of childlessness are influenced by country-level characteristics, which serve as the broader context shaping the psychological well-being of parents and nonparents. One key country-level factor that may enhance the benefits of parenthood is the presence of strong filial elder-care norms. These norms can lead to higher levels of support for aging parents from adult children (Kalmijn and Saraceno 2008), which in turn increases older parents’ SWB (Pinquart and Sörensen 2000). Beyond the tangible support received, filial elder-care norms can also affect life satisfaction by fostering a sense of perceived social support, which refers to the expectation that family members and friends provide support during times of need (Zimet et al. 1988). Research on social support and life satisfaction suggests that both actual received support and perceived support contribute to SWB (Siedlecki et al. 2014). Unlike actual received support, perceived support may also affect the SWB of individuals not currently reliant on filial care and support, as it serves as a reassurance that support will be available when needed. Previous cross-country studies have not explicitly investigated the association between elder-care norms and parental SWB. However, Mair (2013) explored whether national familistic culture moderates the association between parenthood and various indicators of SWB but found no statistically significant differences between countries with low and high levels of familistic norms. Despite Mair’s (2013) finding on parents’ SWB, the overall positive effects of received and perceived social support on overall well-being have been extensively documented (e.g., Pinquart and Sörensen 2000; Siedlecki et al. 2014). Therefore, I expect that the life satisfaction gap between parents and nonparents is more pronounced in countries with strong elder-care norms (Hypothesis 1).

Another country-level factor that may shape the benefits of parenthood is the level of economic vulnerability among older people. In countries where economic vulnerability is high, older parents may derive greater benefits from parenthood than their counterparts in more affluent nations (Grundy 2006) as, in the former, adult children are more likely to provide financial support to their parents than in the later (Brandt 2013; Deindl and Brandt 2011). In addition to this effect, parenthood can serve as a potential safety net that protects against future hazards, thereby also improving the life satisfaction of those who are currently economically secure. Previous research has not explicitly examined the role of economic vulnerability in the association between parenthood and SWB. However, Mair (2013) found that the association between parenthood and SWB is stronger in countries with limited pensions than in countries with generous pensions. This suggests that older individuals derive greater benefits from parenthood in contexts where the economic situation for the elderly is relatively precarious. Considering these arguments and empirical findings, I expect that the association between parenthood and life satisfaction is stronger in counties with high levels of economic vulnerability among older people (Hypothesis 2).

The psychological costs of childlessness might be influenced by the strength of pronatalist norms within a country. Pronatalist norms emphasize the desirability of having children and promote the idea that parenthood enhances individual and societal well-being. At the same time, these norms often convey the notion that childless individuals are avoiding social responsibility and behaving selfishly (Letherby 2002). Although pronatalist norms have weakened in recent decades (Inglehart et al. 2017), parenthood continues to be socially institutionalized as a normative life event in many countries (Connidis 2001; Morgan and King 2001; Schoen et al. 1997). Departures from such social expectations can result in disapproval and social stigma, which may negatively impact SWB (Kaiser and Major 2004).

Although the hypothesis that pronatalist norms shape the SWB of parents and childless individuals is extensively discussed in the literature (e.g., Koropeckyj-Cox et al. 2007), few studies have examined this hypothesis for older individuals. Focusing on childless individuals only, Tanaka and Johnson (2016) found that those living in countries with strong pronatalist norms experience lower SWB than childless individuals in countries with weak pronatalist norms. Huijts et al. (2013) found that strong pronatalist norms are associated with fewer depressive symptoms among younger parents with resident children, but did not observe such gaps between (older) empty-nest parents and their childless counterparts. Given this somewhat mixed evidence, I base my hypothesis on the theoretical arguments outlined above and expect that the association between parenthood and life satisfaction will be more pronounced in countries with strong pronatalist norms (Hypothesis 3).

Contextual factors may not independently influence the benefits of parenthood and costs of childlessness but may interact in shaping these outcomes. Specifically, it is plausible that social norms toward filial elder-care and the risk of economic vulnerability among older individuals interact in moderating the association between parental status and life satisfaction. Strong filial elder-care norms might be particularly important for the life satisfaction of parents in countries where older people face high levels of economic vulnerability (Deindl and Brandt 2017). Therefore, elder-care norms can be expected to shape the life satisfaction of parents and childless individuals in countries with high levels of economic vulnerability to a greater extent than in with lower levels. Conversely, in countries with low levels of economic vulnerability, such norms may have only a marginal impact on the SWB of older people, as reliance on care and support from children is less critical. Therefore, I expect that the association between parenthood and life satisfaction is greatest in countries with high levels of economic vulnerability among older people and strong filial elder-care norms and smallest in countries with low economic vulnerability and weak filial care norms (Hypothesis 4).

Previous research has often suggested that women are more strongly affected by childlessness than men due to gendered social expectations (Koropeckyj-Cox 2002; Simon 1995). However, the findings in this regard are mixed. While some studies indicate that the impact of parenthood on SWB is stronger for women (e.g., Hansen et al. 2009; Koropeckyj-Cox 1998), others suggest that parenthood has stronger effects for men (e.g., Buber and Engelhardt 2008; Dykstra and Wagner 2007; Huijts et al. 2013; Zhang and Hayward 2001). To explore potential gender differences, I conducted a separate analysis stratified by gender. Given the conflicting results of previous studies, I examined gender differences without specific expectations.

## Methods

### Data

My analysis was based on data from the European Social Survey (ESS), a multi-country, cross-sectional time series survey that provides information on life satisfaction and socio-demographic characteristics (www.europeansocialsurvey.org). Conducted biannually since 2001/02, the ESS includes data from 39 countries and currently spans 11 rounds. For this analysis, I focused on Rounds 2–11 (2004–2023) due to the unavailabilty of macro-level indicators for 2002. Additionaly, I excluded five countries from the analysis (Israel, Russia, Ukraine, Kosovo, and Albania) because of missing data on the macro-level indicators required for this study. Finally, I removed data from Turkey (*n* = 338) and Romania (*n* = 515) because of unrealistically high percentages of childless individuals in the ESS data (Turkey: 47%, Romania: 63%). The final sample included 32 countries (see Table 1). As this study focused on the life satisfaction of parents and nonparents in older age, I limited my analysis to respondents aged 60 years and older and excluded 816 individuals who reported having children younger than 18 years of age. After removing 5.5% of observations due to missing data, the final sample comprised 114,513 individuals. The country-specific sample size ranged from 315 in Luxembourg to 9,187 in Germany.

**Table 1:** Distribution of life satisfaction, pronatalist norms, filial elder-care norms, and economic vulnerability

	Life satisfaction		Pronatalist	Filial care	Economic	N of
	Nonparents	Parents	norms^1^	norms^1^	vulnerabilty^2^	cases
Austria	7.54	7.75	2.56	3.80	14.2	4,478
Belgium	7.40	7.50	1.94	4.17	16.9	2,258
Bulgaria	3.98	4.42	3.90	4.30	41.2	5,234
Croatia	5.90	6.50	2.61	4.35	30.9	2,298
Cyprus	6.67	7.18	3.57	4.65	23.1	1,136
Czech Republic	6.27	6.49	3.29	4.15	14.5	4,310
Denmark	8.61	8.70	2.15	2.34	9.3	2,554
Estonia	5.88	6.72	2.93	4.08	41.8	5,422
Finland	7.92	8.11	2.05	2.78	13.0	6,545
France	6.40	6.38	2.22	4.44	10.3	5,197
Germany	7.41	7.46	2.66	3.83	20.0	9,187
Great Britain	7.36	7.50	2.16	3.11	21.4	5,268
Greece	5.38	5.74	3.31	4.59	22.3	2,063
Hungary	5.82	5.79	2.95	4.33	15.7	4,988
Iceland	7.65	8.20	2.04	2.92	5.7	793
Ireland	7.20	7.60	2.47	3.44	21.4	2,910
Italy	6.47	6.72	3.66	4.45	20.5	2,671
Latvia	5.99	5.88	2.94	4.14	44.5	1,263
Lithuania	5.42	5.57	2.95	4.44	40.7	4,494
Luxembourg	8.19	8.13	2.36	3.91	10.5	315
Montenegro	6.24	7.04	2.77	4.65	17.5	489
Netherlands	7.71	7.80	1.76	2.96	13.7	5,009
North Macedonia	6.00	6.09	3.54	4.25	36.4	512
Norway	7.85	8.09	1.76	2.67	7.3	3,863
Poland	6.24	6.61	2.86	4.14	18.9	3,636
Portugal	5.23	5.61	3.24	4.25	21.2	5,894
Serbia	5.47	5.71	2.84	4.44	30.8	1,290
Slovakia	5.87	6.17	2.94	4.72	11.7	3,590
Slovenia	6.69	6.71	2.78	4.10	19.6	3,438
Spain	6.92	7.15	2.76	4.72	17.9	4,584
Sweden	7.72	8.01	1.72	2.53	14.0	5,251
Switzerland	8.36	8.40	2.48	3.51	21.0	3,573
Overall mean	6.87	6.86	2.70	3.91	21.0	114,513

^1^Data extracted from Wave 4 of the European Value Survey (2008). ^2^Data extracted from the database “Persons at risk of poverty or social exclusion by age and sex” (Eurostat 2024a, b). Values refer to 2010

### Outcome variable: life satisfaction

The outcome variable of all analyses was individual life satisfaction. Life satisfaction is a key component of overall SWB (Proctor 2014) and is usually conceptualized as the outcome of a “cognitive evaluation of one’s life” (Diener 1984, p. 550). In the ESS, life satisfaction was assessed using the item, “All things considered, how satisfied are you with your life as a whole nowadays?” on an 11-point scale (0 = extremely dissatisfied to 10 = extremely satisfied).

### Key predictor variable: parental status

The primary explanatory variable at the individual level was parental status, which distinguished between parents and childless individuals. I created an indicator for parenthood by combining two types of information. Information on co-residing (adult) children was collected through questions about household composition. Information on children not living in the respondent’s household was obtained through the question, “Have you ever had any children of your own, step-children, adopted children, foster children, or a partner’s children living in your household?” Respondents were classified as parents if they co-resided with an (adult) child or indicated that they had ever lived with a child. Table 1 presents the country-specific means of life satisfaction for parents and nonparents.

### Macro-level variables

The explanatory variables at the country level were pronatalist norms, social norms toward filial elder-care, and level of economic vulnerability among older people. The normative context can be indicated by descriptive and injunctive norms. Descriptive norms are typical patterns of behavior, generally accompanied by the expectation that people will behave according to these patterns. Injunctive norms, in contrast, describe prescriptive rules or normative expectations specifying how people should act (Kitts and Chiang 2008). In this study, I measured the normative context by a country’s injunctive norm. Injunctive pronatalist and elder-care norms were indicated by aggregated personal attitudes obtained from the European Value Survey (EVS) conducted in 2008 and 2017.

Pronatalist norms were indicated by agreement or disagreement with the statement, “It is a duty toward society to have children,” and filial elder-care norms were measured with the statement, “Adult children have the duty to provide long-term care for their parents.” The response sets of both items ranged from strongly agree (1) to strongly disagree (5). I reversed both items and created country-level indicators by calculating the mean of each item for each country. When creating the indicator for filial elder-care norms, I used only information from respondents younger than 60 years to ensure a focus on younger individuals’ willingness to provide filial elder-care rather than older individuals’ expectation of receiving it. To accommodate changes in social norms over time, I calculated the country-specific means separately for 2008 and 2017 and assigned the means obtained from the 2008 EVS to all data in the sample collected between 2004 and 2013 and the means obtained from the 2017 EVS to all data collected between 2014 and 2023. Higher values in the indicator variables indicated stronger pronatalist and filial elder-care norms, respectively.

Economic vulnerability is understood as “heightened risk of multidimensional deprivation” (Whelan and Maître 2008: 638) and includes poverty and other forms of social exclusion. A measure of a country’s level of economic vulnerability is the so-called AROPE (“At risk of poverty and social exclusion”) indicator provided by Eurostat, which reflects the percentage of persons either at risk of poverty or severely materially and socially deprived (Zins 2020). To indicate the level of economic vulnerability of older people within a country, I used the yearly AROPE rate for persons aged 60 years and older, which has been published by Eurostat since 2003. The AROPE rates for 2015–2023 are available in the database ilc_peps01n (Eurostat 2024a); the rates for earlier years (2004–2014) were obtained from the database ils_peps01 (Eurostat 2024b). I matched the yearly data provided by Eurostat for each country to the specific country-years of the ESS data on the basis of the year of the interview. To control for country differences in wealth, I also included a variable reflecting the logarithm of the gross domestic product per capita (log GDP). In the analysis, all country-level indicators were z-standardized. In general, the correlations between macro-level variables were rather high. Strong correlations (*r*^2^  = 0.60) existed between pronatalist norms and elder-care norms (*r*^2^ = 0.66), pronatalist norms and economic vulnerability (*r*^2^ = 0.61), GDP and pronatalist norms (*r*^2^ = − 0.60), and GDP and elder-care norms (*r*^2^ = − 0.65).

### Control variables

All regression models controlled for partnership status, employment status, educational level, age, health status, and economic hardship. Partnership status was captured by a variable indicating whether the respondents were married (or living with a partner). Employment status was measured by a variable indicating whether the respondents were gainfully employed, unemployed, or out of the labor force (e.g., homemakers, retired). Educational level was captured by distinguishing between respondents with low (lower secondary education or less), medium (upper secondary education), and high (post-secondary or tertiary education) educational attainment. I controlled for age using linear and squared terms, as life satisfaction and age are associated in a nonlinear fashion (Hansen and Blekesaune 2022). Health status was rated by respondents on a five-point scale, ranging from 1 (very good) to 5 (bad). Economic hardship was measured by asking how respondents feel about their present household income. I categorized as experiencing economic hardship all those respondents who reported finding it difficult or very difficult to live on their present income.

Controlling for health and economic hardship seems crucial for identifying the associations between filial elder-care norms and the level of economic vulnerability with parents’ life satisfaction. As mentioned earlier, strong elder-care norms may prompt adult children to provide care and support to their aging parents, thereby improving physical and mental health and ultimately life satisfaction. Similarly, children may be more likely to provide financial support to their parents in countries with high economic vulnerability than in countries with low levels of economic vulnerability, which may enhance life satisfaction. Consequently, individual health and economic well-being may mediate the impact of having children on levels of life satisfaction. Including indicators for the health status and economic circumstances of older individuals can adjust for these effects and help to identify associations between macro-level factors and parents’ life satisfaction.

### Method

For the main analysis, I estimated cross-classified multilevel models (Rabe-Hesketh and Skrondal 2012; Schmidt-Catran and Fairbrother 2016) with two levels: country and year of the interview. By doing so, I took into account that individuals are nested in two higher-level contexts. Because statistical causality is difficult to establish in cross-sectional research, the results presented here need to be interpreted as statistical associations rather than causal effects. To test whether the correlation between parenthood and life satisfaction is moderated by the national context, I included cross-level interactions between the context indicators and the parental status indicator. All models included a random slope for parental status, allowing the effect of the parenthood indicator to vary between contexts.

As Stegmueller (2013, p. 758) noted, the estimates for cross-level interactions and their standard errors tend to be biased downward in linear models when the sample size at level 2 is small. To avoid overloading the model with cross-level interactions, most models included only one cross-level interaction at a time between parenthood and the macro-level indicators of interest. All analyses were conducted using Stata 17.

## Results

Model 1 in Table 2 sets out the base model for the multi-level analysis. As expected, parents were significantly more satisfied with their lives than nonparents (*b* = 0.078, *p* < 0.01). To explore gender differences, I estimated Model 1 separately for women and men (results not reported in a table). The association between parenthood and life satisfaction was slightly stronger for men (*b* = 0.078, *p* < 0.01) than for women (0.068, *p* < 0.01). However, a formal test of the gender interaction, conducted by adding an interaction term to Model 1, revealed no significant gender differences in the association between parenthood and life satisfaction.

**Table 2:** Unstandardized regression coefficients predicting life satisfaction

	Model 1		Model 2		Model 3	
	b	*SE*	b	*SE*	b	*SE*
Parent	0.078**	0.016	0.092**	0.020	0.090**	0.020
Woman	0.145**	0.012	0.144**	0.012	0.145**	0.012
Age	0.060**	0.013	0.060**	0.013	0.060**	0.013
Age squared/100	− 0.001**	0.000	− 0.001**	0.000	− 0.001**	0.000
Married or cohabiting	0.363**	0.013	0.362**	0.012	0.362**	0.012
Education^1^						
- Upper secondary	0.060**	0.014	0.060**	0.014	0.060**	0.014
- Tertiary	0.100**	0.032	0.100**	0.032	0.100**	0.033
Employment status^2^						
- Unemployed	− 0.572**	0.052	− 0.572**	0.052	− 0.572**	0.052
- Out of labor force	0.018	0.019	0.017	0.019	0.017	0.019
Economically deprived	− 1.088**	0.015	− 1.088**	0.015	− 1.088**	0.015
Health^3^						
- Good	− 0.477**	0.021	− 0.447**	0.021	− 0.477**	0.021
- Fair	− 1.057**	0.022	− 1.057**	0.022	− 1.058**	0.022
- Bad	− 1.901**	0.026	− 1.899**	0.026	− 1.899**	0.026
- Very bad	− 2.849**	0.040	− 2.848**	0.040	− 2.848**	0.040
*Macro-level indicators*						
Elder-care norms	− 0.052	0.055	− 0.221*	0.088	− 0.053	0.055
Economic vulnerability	0.057	0.041	0.071	0.041	− 0.001	0.047
Pronatalist norms	− 0.240**	0.064	− 0.186**	0.069	− 0.238**	0.064
GDP (log)	0.359**	0.057	0.329**	0.053	0.365**	0.062
Elder-care norms*Parent			0.007	0.023		
Economic vulnerability*Parent					0.065*	0.026
GDP (log)*Parent			− 0.028	0.030	0.001	0.028
Constant	7.661**	0.082	4.812**	0.490	4.764**	0.492
*Variance components*						
Var RI Country	0.136	0.042	0.134	0.038	0.134	0.042
Var RI Country-year	0.040	0.005	0.052	0.011	0.051	0.011
Var RI Year	0.011	0.015	0.004	0.005	0.012	0.017
Var Level-1	3.744	0.015	3.741	0.015	3.741	0.016
Var RS Parent			0.020	0.008	0.018	0.007
Cov (RS, RI Country-year)			− 0.015	0.008	− 0.014	0.008

^1^Reference group: less than upper secondary education; ^2^reference group: gainfully employed; ^3^reference group: very good. *RI* random intercept. *RS* random slope

N of countries: 32; N of country-years: 202; N of observation: 114,513

*Significance levels*: *p < 0.05, **p < 0.01

Among the macro-level indicators, elder-care norms and economic vulnerability showed no correlation with life satisfaction, while pronatalist norms were negatively associated with life satisfaction. This negative association may be attributed to correlations between pronatalist norms and other macro-level factors such as individualism (Liefbroer et al. 2014), which is known to influence life satisfaction (Oishi et al. 2009).

In the next step, I examined whether the association between parental status and life satisfaction is shaped by context factors. To investigate whether elder-care norms moderate the association between parenthood and life satisfaction, I included a cross-level interaction between parental status and elder-care norms in Model 2.

**Fig. 1:**
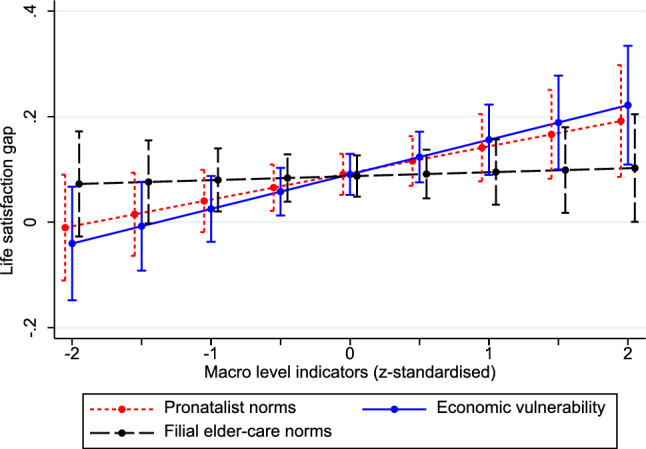
Predicted life satisfaction advantage of parents compared to nonparents from Models 2 to 4 in Tables 2 and 3

Contrary to my expectations expressed in Hypothesis 1, the cross-level interaction between parental status and elder-care norms was very small and statistically insignificant (*b* = 0.007, *p* > 0.05). To illustrate this interaction effect (and others), I calculated the life satisfaction gap between parents and nonparents for varying values of the macro-level indicators (Fig. 1). Figure 1 shows that the life satisfaction gap between parents and non-parents was almost identical in contexts with strong and weak filial elder-care norms.

Model 3 (Table 2) examined whether the association between parenthood and life satisfaction was affected by the level of economic vulnerability among older people. The positive interaction coefficient (*b* = 0.065, *p* < 0.05) aligns with Hypothesis 2 that the association between parenthood and life satisfaction varies between countries with high and low levels of economic vulnerability. In countries with high levels of economic vulnerability (indicator is one standard deviation above mean), the life satisfaction gap between parents and nonparents was 0.156 points (*p* < 0.01), whereas in countries with low levels (indicator is one standard deviation below the mean), the life satisfaction gap was 0.025 points (*p* > 0.05).

Model 4 in Table 3 explored whether the life satisfaction gap between parents and nonparents varied depending on the strength of pronatalist norms. The positive interaction (*b* = 0.050, *p* < 0.01) supports Hypothesis 3 that the life satisfaction gap is significantly smaller in countries with weak pronatalist norms compared to those with strong pronatalist norms. As illustrated in Fig. 1, in countries in which the macro-level indicator was one standard deviation above the mean, the life satisfaction gap amounted to 0.141 (*p* < 0.01). Conversely, in countries in which the macro-level indicator was one standard deviation below the mean, the gap decreased to 0.040 (*p* > 0.05).

**Table 3:** Unstandardized regression coefficients predicting life satisfaction

	Model 4		Model 5		Model 6	
	b	*SE*	b	*SE*	b	*SE*
Parent	0.090**	0.020	0.091**	0.020	0.051*	0.022
Woman	0.145**	0.012	0.145**	0.012	0.146**	0.012
Age	0.060**	0.013	0.058**	0.013	0.058**	0.013
Age squared/100	− 0.001**	0.000	− 0.002**	0.000	− 0.001**	0.000
Married or cohabiting	0.361**	0.013	0.361**	0.012	0.361**	0.012
Education^1^						
- Upper secondary	0.060**	0.014	0.031*	0.014	0.031*	0.014
- Tertiary	0.100**	0.033	0.145**	0.018	0.145**	0.032
Employment status^2^						
- Unemployed	− 0.573**	0.052	− 0.563**	0.052	− 0.562**	0.052
- Out of labor force	0.017	0.019	0.026	0.019	0.026	0.019
Economically deprived	− 1.088**	0.015	− 1.082**	0.015	− 1.082**	0.015
Health^3^						
- Good	− 0.478**	0.021	− 0.473**	0.021	− 0.474**	0.021
- Fair	− 1.058**	0.022	− 1.050**	0.022	− 1.050**	0.022
- Bad	− 1.899**	0.026	− 1.891**	0.026	− 1.891**	0.026
- Very bad	− 2.848**	0.040	− 2.841**	0.040	− 2.841**	0.040
*Macro-level indicators*						
Elder-care norms	− 0.055	0.055	− 0.187*	0.090	− 0.056	0.062
Economic vulnerability	0.055	0.039	0.027	0.048	0.035	0.048
Pronatalist norms	− 0.278**	0.067	− 0.231**	0.007	− 0.288**	0.068
GDP (log)	0.370**	0.063	0.297**	0.054	0.349**	0.065
Elder-care norms*Parent			− 0.033	0.029	0.004	0.031
Economic vulnerability*Parent			0.051^**+**^	0.028	0.023	0.028
Pronatalist norms *Parent	0.050*	0.024	0.053^**+**^	0.031	0.061*	0.028
Care norms*econ. vulner					− 0.114*	0.048
Care norms* Econ. vulner.*Parent					0.119**	0.030
GDP (log)*Parent	0.001	0.030	0.005	0.032	0.017	0.034
Constant	4.764**	0.492	4.880**	0.491	4.871**	0.493
*Variance components*						
Var RI Country	0.134	0.042	0.136	0.039	0.137	0.044
Var RI Country-year	0.049	0.011	0.052	0.011	0.047	0.010
Var RI Year	0.013	0.017	0.004	0.005	0.012	0.017
Var Level-1	3.741	0.016	3.740	0.015	3.740	0.015
Var RS Parent	0.018	0.008	0.018	0.007	0.014	0.007
Cov (RS, RI Country-year)	− 0.013	0.008	− 0.014	0.008	− 0.010	0.007

^1^Reference group: less than upper secondary education; ^2^reference group: gainfully employed; ^3^reference group: very good. *RI* random intercept. *RS* random slope

N of countries: 32; N of country-years: 202; N of observation: 114,513

*Significance levels*: ^**+**^*p* < 0.1, *p < 0.05, **p < 0.01

Models 2–4 include only one cross-level interaction between parenthood and a macro-level indicator of interest. As discussed by Blackwell and Olson (2022), such single interaction models are prone to omitted interaction bias, which occurs when the variable of interest (in this case parenthood) interacts with other covariates (in this case other macro-level indicators). To test the robustness of these findings, I estimated a model that included all cross-level interactions. The resulting pattern, shown in Model 5 (Table 3), aligns with the results shown in Models 2–4, although the coefficients of the cross-level interactions partly decreased in size and were only marginally statistically significant. In models stratified by gender (results not shown in a table), the cross-level interactions between parental status and the macro-level indicators were similar in magnitude for both men and women, though not always statistically significant. Overall, these models suggest that the country-level factors examined affect the life satisfaction of men and women to a comparable extent.

In summary, the findings from Models 2 to 5 align with the expectation that the association between parenthood and life satisfaction is stronger in countries with a high level of economic vulnerability among older individuals (Hypothesis 2) and strong pronatalist norms (Hypothesis 3) than in countries with a low level of economic vulnerability and weak pronatalist norms. Contradicting Hypothesis 1, the findings did not indicate that the life satisfaction gap between parents and nonparents correlated with a country’s level of elder-care norms.

In the final stage of analysis, I explored whether the association between parenthood and life satisfaction was jointly associated with elder-care norms and the level of economic vulnerability among older people. As discussed above, strong elder-care norms may be particularly important for parents’ life satisfaction in contexts where older people face high levels of economic vulnerability. Conversely, in countries with low levels of economic vulnerability, elder-care norms may have only a weak impact on parents’ life satisfaction. In other words, I hypothesized that the interaction between parenthood and elder-care norms varies across levels of economic vulnerability. To test this, I estimated a model that included a three-way interaction term along with all two-way interactions of parental status with elder-care norms, pronatalist norms, and economic vulnerability (Model 6 in Table 3). The three-way interaction was statistically significant (b = 0.119, *p* < 0.01), indicating that elder-care norms and the economic vulnerability of older people jointly moderated the association between parental status and life satisfaction.

To facilitate the interpretation of the three-way interaction, I calculated the predicted levels of life satisfaction for both parents and nonparents in countries with strong/weak elder-care norms and low/high level of economic vulnerability, respectively (Table 4). In line with Hypothesis 4, the life satisfaction gap between parents and nonparents was largest in countries with high levels of economic vulnerability and strong elder-care norms (*b* = 0.20, *p* < 0.01). Conversely, in countries with high levels of economic vulnerability and weak elder-care norms, as well as in those with low levels of economic vulnerability and strong elder-care norms, parents did not report higher levels of life satisfaction than nonparents. However, contradicting Hypothesis 4, a large life satisfaction gap between parents and non-parents (*b* = 0.14, *p* < 0.01) also appeared in countries with low levels of economic vulnerability and weak elder-care norms.

**Table 4:** Predicted levels of life satisfaction from Table 2, Model 5

	Parents	Non-parents	LS gap between parents and nonparents
(1) Low economic vulnerability^1^ & weak elder-care norms^1^	6.86	6.72	0.14**
(2) Low economic vulnerability^1^ & strong elder-care norms^2^	6.75	6.83	− 0.08
(3) High economic vulnerability^2^ & weak elder-care norms^1^	6.97	7.02	− 0.05
(4) High economic vulnerability^2^ & strong elder-care norms^2^	6.88	6.68	0.20**

^1^Value of the macro-level indicator is 1 SD below the grand mean. ^2^Value of the macro-level indicator is 1 SD above the grand mean. *LS* life satisfaction

*Significance levels*: *p < 0.05, **p < 0.01

 Partially supporting Hypothesis 1, the results suggest that elder-care norms shape the life satisfaction gap between parents and nonparents in counties with high levels of economic vulnerability (but not in those with low levels). In countries with strong elder-care norms (and high economic vulnerability), the life satisfaction gap was substantial and statistically significant (*b* = 0.20, *p* < 0.01), whereas in countries with weak elder-care norms, it was small and statistically insignificant (*b* = − 0.05, *p* > 0.05). The difference between these two life satisfaction gaps was also statistically significant (*b* = 0.25, *p* = 0.016, not reported in Table 4).

### Robustness checks

Model 6 in Table 3 may have produced biased estimates due to the inclusion of six cross-level interactions and six variance parameters. To test the robustness of this model, I estimated an additional model by categorizing the 32 countries into four groups based on the grand means of elder-care norms and economic vulnerability. These four country groups were defined as follows: strong elder-care norms and low economic vulnerability, strong elder-care norms and high economic vulnerability, weak elder-care norms and low economic vulnerability, and weak elder-care norms and high economic vulnerability. Model 1 in Table 6 (in appendix) shows no significant differences in life satisfaction between these four country groups. The overall association between parenthood and life satisfaction was positive and statistically significant (*b* = 0.090, *p* < 0.01). In Model 2, I included interactions between the country group variable and parental status. The results indicate that the association between parenthood and life satisfaction was significantly stronger in countries with high levels of economic vulnerability and strong filial elder-care norms (*b* = 0.175, *p* < 0.01) than in the reference group (countries with low levels of economic vulnerability and strong elder-care norms). In the two other country groups, the life satisfaction gaps between parents and nonparents did not differ from the gap in the reference countries. On basis of these coefficients, I calculated the predicted level of life satisfaction for parents and nonparents in each country group (Table 7 in appendix). These estimates align closely with the results presented in Table 3, indicating that the life satisfaction gap between parents and non-parents was strongest in countries with high levels of economic vulnerability and strong elder-care norms.

In an additional robustness check, I tested whether the heterogeneous effects of the macro-level factors on life satisfaction were influenced by micro-level characteristics associated with parenthood, such as partnership status, health, or financial deprivation. To do so, I added interactions between parenthood and all micro-level variables to the model. The results, presented in Table 8 (in appendix), reveal no statistically significant interactions, except for the interaction between parenthood and having upper secondary-level educational attainment. Importantly, including these micro-level interactions did not significantly alter the cross-level interaction effects. They remained consistent with those reported in Tables 2 and 3.

## Discussion and conclusion

Family research commonly views parenthood as a key predictor of the well-being of older individuals (see, Hansen 2012). However, empirical findings have been inconsistent, with studies showing positive, negative, or no associations between parental status and life satisfaction. A common explanation for these conflicting findings is that the impact of parenthood on older individuals’ SWB strongly depends on societal context. The aim of the present study was to identify macro-level contexts in which parents experience a life satisfaction advantage over nonparents and those in which they do not.

My analysis showed that, across the surveyed European countries, parents generally reported higher life satisfaction than nonparents. However, the extent of this life satisfaction gap varied significantly across contexts. Contrary to my expectations expressed in Hypothesis 1, the results did not indicate that the life satisfaction gap is consistently greater in countries with strong filial elder-care norms compared to countries with weak elder-care norms. Nevertheless, in countries with high levels of economic vulnerability, where older people are most dependent on informal support, the presence of strong filial elder-care norms was associated with larger life satisfaction gaps compared to weak elder-care norms. This finding provides partial support for Hypothesis 1. I will revisit this result in the discussion of Hypothesis 4.

Supporting Hypothesis 2, my analysis indicates that the life satisfaction gap between parents and nonparents is larger in countries where older individuals face high levels of economic vulnerability than in those with low levels of economic vulnerability. This finding is consistent with previous studies examining the role of related macro-level indicators for older people’s SWB. For instance, Mair (2013) found that the advantages of parenthood are greater in countries with low pension spending than in those with high pension spending. Likewise, Albertini and Mencarini (2014) proposed that parenthood is more important to the SWB of older people in societies where transfers and public services to older people are limited.

My analysis also supports Hypothesis 3 that the life satisfaction gap is larger in countries with strong pronatalist norms than in those with weak pronatalist norms. This finding extends and refines the analyses by Huijts et al. (2013), who observed that pronatalist norms shape the life satisfaction gap between (younger) parents with resident children and childless individuals, but did not detect any such gap between (older) empty-nest parents and their childless counterparts.

Finally, my findings indicate that social context factors do not independently affect the life satisfaction gap between parents and nonparents; rather, they affect it by interacting with each other. However, my results only partly support Hypothesis 4, which suggests that the life satisfaction gap is greatest in countries with high levels of economic vulnerability and strong filial elder-care norms and smallest in countries with low economic vulnerability and weak filial care norms. In line with that hypothesis, I found the life satisfaction gap between parents and nonparents to be largest in countries with high levels of economic vulnerability and strong elder-care norms. However, in contrast to Hypothesis 4, a strong association between parenthood and life satisfaction also appeared in contexts with low levels of economic vulnerability and low social care norms.

While this finding is somewhat unexpected, it aligns with the arguments of Neuberger and Preisner (2018), who distinguished between the supportive and the emotional benefits of parenthood. They suggest that supportive benefits are most significant in contexts in which older individuals rely on informal support, whereas emotional benefits are greatest when the parent–child relationship is free from filial support obligations, allowing parents to derive life satisfaction from higher levels of emotional closeness and connectedness with their children. Consequently, in countries with strong elder-care norms and high economic vulnerability parents are most reliant on instrumental support and may benefit predominantly from parenthood through the provision of such support. In contrast, in countries with weak elder-care norms and low economic vulnerability the parent–child relationship is most unburdened care obligations and parents may primarily gain from the emotional aspects of parenthood. These findings suggest that because parenthood can have both supportive and emotional benefits, the life satisfaction advantage of parenthood, as well as the disadvantage of childlessness, is likely shaped not by a single context characteristic nor by several characteristics operating independently of each other, but rather by the interplay of a variety of context factors.

It is important to note that the macro-level characteristics considered in this study are dynamic and have undergone substantial changes over time, which may alter the association between parenthood and life satisfaction. The data on economic vulnerability used in this study indicate that the percentage of older individuals experiencing economic vulnerability decreased by 0.3% points per year. Likewise, the intergenerational shift toward individual-choice norms (Inglehart et al. 2017) has weakened pronatalist norms, although the macro-level indicators used in this study do not show notable changes in pronatalist norms between 2008/10 and 2017/21. These broader societal trends suggest that some of the benefits of parenthood have diminished over time and are likely to continue declining. However, it remains an open question whether these trends will result in a progressively weaker association between parenthood and life satisfaction or whether the emotional benefits of parenthood will become increasingly important as the relevance of the instrumental benefits diminishes.

This study is not without limitations. First, like almost all studies on the association between parental status and SWB in old age, this study relied on cross-sectional data and therefore could not address issues of causality. Specifically, the research design does not account for selection processes, which occur when individuals with high levels of life satisfaction are more likely to become parents than individuals with lower life satisfaction. However, previous studies have found that SWB has little effect on the transition to parenthood after other predictors of fertility are considered (Le Moglie et al. 2015; Perelli-Harris 2006). Hansen (2012: 40) also concludes that it “seems unlikely that reverse causation or unobserved third factors (e.g., personality traits) are accounting for cross-sectional associations between parental status and well-being.”

Second, it must be acknowledged that the contextual factors examined in this study may correlate with other contextual factors that have not been considered. For example, research has shown that countries with low levels of informal social support often have extensive social policies, and vice versa (Bolin et al. 2008; Bonsang 2009). Although this type of limitation applies to almost all cross-country studies, it is important to remember that cross-country differences regarding a specific context factor under study may be influenced by a related but unexamined factor. A related limitation is that the data for this study did not allow for distinguishing between macro-level social norms and individual-level personal norms. Individuals in countries with strong pronatalist norms typically hold stronger pronatalist values than those in countries with weaker pronatalist norms. Because the data used in this study did not provide information on personal norms, the present study cannot clarify whether the greater life satisfaction penalty for childless individuals in pronatalist countries arises from social norms that foster feeling of disapproval and social deviation or from stronger pronatalist attitudes held by childless individuals themselves. However, previous studies suggest that social norms affect individuals’ life satisfaction regardless of their personal norms. For instance, Stavrova and Fechtenhauer (2014) examined the impact of social and personal norms regarding childbearing in cohabiting unions on parents’ life satisfaction. They found that cohabiting parents reported lower life satisfaction in countries with strong norms against childbearing out of wedlock, even when they did not personally hold these norms.

Despite these limitations, this study contributes to the ongoing debate on whether, and under what circumstances, childlessness has negative consequences for the SWB of older people. It corroborates the notion that context factors shape the benefits of parenthood and costs of childlessness, and that cross-country differences in these factors contribute to cross-country differences in the life satisfaction gap between parents and nonparents.

## Data Availability

The data from the European Social Suvey are available at https://www.europeansocialsurvey.org. The data from the European Values Survey are available at https://www.gesis.org.

## Appendix

See appendix Tables 5, 6, 7 and 8.

**Table 5:** Descriptive statistics

Variable	Total	Parent	Childless
Life satisfaction (mean)	6.89	6.86	6.87
Female (in %)	55.59	56.09	52.91
Age (in years)	70.72	70.69	70.87
Married/ coh. (in %)	60.42	63.20	45.56
Parent (in %)	84.46		
Employment status (in %)			
(Self-) employed	14.30	14.31	14.21
- Unemployed	1.33	1.23	1.88
- Out of labor force	84.37	84.46	83.91
Education (in %)			
-Lower secondary or less	41.34	71.33	41.38
-Upper secondary	42.26	42.46	41.17
-Post-secondary	16.43	16.21	17.44
Economic deprived (in %)	26.58	26.91	24.82
Health (in %)			
- Very good	9.13	9.08	9.39
- Good	33.69	33.34	35.59
- Fair	40.91	41.18	39.45
- Bad	13.39	13.53	12.60
- Very bad	2.88	2.87	2.97
N	114,513	96,718	17,795

**Table 6:** Unstandardized regression coefficients predicting life satisfaction differentiated by country groups (multi-level regression model)

Variable	Model 1		Model 2	
	b	*SE*	b	*SE*
Low economic vulnerability^1^ & strong elderly care norms^2^	Ref		Ref	
Low economic vulnerability^1^ & weak elderly care norms^1^	0.030	0.085	− 0.057	0.098
High economic vulnerability^2^& strong elderly care norms^2^	− 0.044	0.066	− 0.190*	0.084
High economic vulnerability^2^ & weak elderly care norms^1^	0.146	0.092	0.101	0.107
Parent	0.090**	0.020	0.002	0.037
Low economic vulnerability & weak care norms*Parent			0.105	0.058
High economic vulnerability & strong care norms*Parent			0.175**	0.062
High economic vulnerability & weak care norms*Parent			0.050	0.066
N of countries /country-years	32 / 202		32/202	
Observations	114,513		114,513	

^1^Value of the macro-level indicator is below the grand mean. ^2^Value of the macro-level indicator above the grand mean. All models control for sex, age, education, partnership status, number of children, employment status, log GDP, interaction between log GDP and parenthood, pronatalist norms, and interaction between pronatalist norms and parenthood

*Significance levels*: **p* < 0.05, ***p* < 0.01

**Table 7:** Predicted levels of life satisfaction from Table 6, Model 2

	Parents	Non-parents	LS gap between parents and non-parents
Low economic vulnerability & weak elderly care norms	6.95	6.85	0.10*
Low economic vulnerability & strong elderly care norms	6.91	6.90	0.01
High economic vulnerability & weak elderly care norms	7.06	7.01	0.05
High economic vulnerability & strong elderly care norms	6.89	6.71	0.18**

*Significance levels*: **p* < 0.05, ***p* < 0.01

**Table 8:** Unstandardized regression coefficients predicting life satisfaction

	Model 1		Model 2		Model 3		Model 4		Model 5	
	b	*SE*	b	*SE*	b	*SE*	b	*SE*	b	*SE*
Parent	0.077	0.080	0.076	0.052	0.092	0.080	0.090	0.081	0.045	0.081
Woman	0.171**	0.029	0.172**	0.029	0.172**	0.029	0.175**	0.029	0.172**	0.029
Woman*Parent	− 0.032	0.032	− 0.034	0.032	− 0.033	0.032	− 0.036	0.032	− 0.033	0.032
Age	0.017**	0.002	0.018**	0.002	0.017**	0.002	0.018**	0.002	0.018**	0.002
Age*Parent	0.004	0.003	0.003	0.002	0.004	0.003	0.003	0.003	0.004	0.002
Age squared/100	− 0.043	0.022	− 0.043*	0.022	− 0.043*	0.022	− 0.054*	0.022	− 0.045*	0.022
Age squared*Parent	0.020	0.024	0.020	0.024	0.024	0.020	0.032	0.024	0.022	0.022
Married/cohabiting	0.325**	0.030	0.327**	0.030	0.325**	0.030	0.326**	0.030	0.329**	0.030
Married/coh.*Parent	0.042	0.033	0.040	0.033	0.042	0.033	0.044	0.034	0.038	0.033
Education										
- Upper secondary	0.150**	0.033	0.150**	0.033	0.160**	0.033	0.169**	0.033	0.158**	0.033
- Upper sec.*parent	− 0.104**	0.036	− 0.107**	0.035	− 0.116**	0.036	− 0.124**	0.036	− 0.114**	0.035
- post-secondary	0.120	0.074	0.131	0.074	0.120	0.074	0.109	0.075	0.114	0.074
- Post sec.*parent	− 0.034	0.081	− 0.031	0.081	− 0.018	0.081	− 0.011	0.082	− 0.010	0.081
Employment status^2^										
- Unemployed	− 0.669**	0.112	− 0.666**	0.112	− 0.669**	0.112	− 0.695**	0.112	− 0.670**	0.112
- Unempl.*parent	0.140	0.127	0.140	0.127	0.142	0.127	0.156	0.127	0.142	0.127
- Out of labor force	0.036	0.047	0.034	0.047	0.034	0.047	0.025	0.047	0.031	0.047
- Out of lab.*parent	− 0.021	0.051	− 0.018	0.051	− 0.019	0.051	− 0.009	0.051	− 0.015	0.051
Econ. deprived	− 1.115**	0.038	− 1.114**	0.038	− 1.137**	0.038	− 1.142**	0.038	− 1.113**	0.038
Econ. depr.*Parent	0.076	0.041	0.065	0.041	0.058	0.041	0.064	0.041	0.055	0.041
Health^3^										
- Good	− 0.525**	0.053	− 0.522**	0.053	− 0.518**	0.053	− 0.512**	0.053	− 0.524**	0.053
- good*Parent	0.055	0.058	0.051	0.058	0.046	0.058	0.042	0.058	0.053	0.058
- Fair	− 1.074**	0.053	− 1.071**	0.053	− 1.065**	0.053	− 1.059**	0.053	− 1.072**	0.053
- fair*Parent	0.021	0.058	0.018	0.058	0.010	0.058	0.003	0.058	0.019	0.058
- Bad	− 1.978**	0.065	− 1.978**	0.065	− 1.970**	0.065	− 1.949**	0.065	− 1.977**	0.065
- bad*Parent	0.097	0.070	0.091	0.0 70	0.081	0.070	0.059	0.072	0.092	0.070
- Very bad	− 3.001**	0.098	− 3.001**	0.098	− 3.001**	0.098	− 3.012**	0.098	− 3.012**	0.098
- very bad*Parent	0.185	0.108	0.184	0.108	0.176	0.108	0.195	0.109	0.188	0.108
*Macro-level ind*										
Elder-care norms	− 0.216	0.088	− 0.055	0.055	− 0.057	0.055	− 0.177*	0.089	− 0.045	0.062
Econ. vulnerability	0.071	0.041	0.001	0.047	0.054	0.041	0.030	0.047	0.038	0.048
Pronatalist norms	− 0.188**	0.069	− 0.237**	0.064	− 0.283**	0.067	− 0.246**	0.074	− 0.301**	0.067
Log GDP	0.309**	0.053	0.349**	0.063	0.349**	0.064	0.277**	0.054	0.334**	0.065
Care norms*Parent	− 0.003	0.024					− 0.043	0.028	− 0.010	0.031
Vulnerability*Parent			0.064*	0.026			0.057^+^	0.281	0.020	0.028
Pron. norms*Parent					0.058*	0.025	0.069*	0.0324	0.075**	0.029
Care norms* Vulner									− 0.107*	0.048
Care * Vuln.* Parent									− 0.113**	0.030
Log GDP*Parent	− 0.004	0.031	0.034	0.029	0.031	0.031	0.033	0.033	0.039	0.035
Constant	7.71**	0.104	7.671**	0.104	7.660**	0.107	7.707**	0.104	7.711**	0.108
*Variance comp*										
Var RI Country	0.133	0.038	0.135	0.043	0.131	0.043	0.133	0.043	0.130	0.047
Var RI Country-year	0.051	0.011	0.049	0.011	0.048	0.011	0.048	0.011	0.045	0.010
Var RI Year	0.005	0.006	0.013	0.014	0.014	0.015	0.013	0.015	0.013	0.014
Var Level-1	3.740	0.015	3.740	0.015	3.740	0.016	3.740	0.016	3.740	0.015
Var RS Parent	0.020	0.008	0.018	0.008	0.018	0.008	0.017	0.008	0.013	0.007
Cov (RS, RI Con-yr)	− 0.015	0.008	− 0.013	0.008	− 0.012	0.008	− 0.012	0.008	− 0.008	0.007

^1^Reference group: less than upper secondary education; ^2^reference group: gainfully employed; ^3^reference group: very good. *RI* random intercept. *RS* random slope

N of countries: 32; N of country-years: 202; N of observation: 114,513

*Significance levels*: ^+^*p* < 0.1, **p* < 0.05, ***p* < 0.01
